# Occult HCV Infection: An Unexpected Finding in a Population Unselected for Hepatic Disease

**DOI:** 10.1371/journal.pone.0008128

**Published:** 2009-12-02

**Authors:** Laura De Marco, Anna Gillio-Tos, Valentina Fiano, Guglielmo Ronco, Vittorio Krogh, Domenico Palli, Salvatore Panico, Rosario Tumino, Paolo Vineis, Franco Merletti, Lorenzo Richiardi, Carlotta Sacerdote

**Affiliations:** 1 Unit of Cancer Epidemiology, C.E.R.M.S., University of Turin, Turin, Italy; 2 Center for Oncologic Prevention, University of Turin, Turin, Italy; 3 Epidemiology Unit, National Cancer Institute, Milan, Italy; 4 Molecular and Nutritional Epidemiology Unit, Cancer Research and Prevention Institute - ISPO, Florence, Italy; 5 Department of Clinical and Experimental Medicine, Federico II University, Naples, Italy; 6 Cancer Registry, “Civile M.P. Arezzo” Hospital, Ragusa, Italy; 7 Imperial College London, and University of Turin, Turin, Italy; Karolinska Institutet, Sweden

## Abstract

**Background:**

Occult Hepatitis C virus (HCV) infection is a new pathological entity characterized by presence of liver disease and absence or very low levels of detectable HCV-RNA in serum. Abnormal values of liver enzymes and presence of replicative HCV-RNA in peripheral blood mononuclear cells are also observed. Aim of the study was to evaluate occult HCV occurrence in a population unselected for hepatic disease.

**Methodology/Principal Findings:**

We chose from previous epidemiological studies three series of subjects (n = 276, age range 40–65 years) unselected for hepatic disease. These subjects were tested for the presence of HCV antibodies and HCV-RNA in plasma and in the peripheral blood mononuclear cells (PBMCs) by using commercial systems. All subjects tested negative for HCV antibodies and plasma HCV-RNA and showed normal levels of liver enzymes; 9/276 patients (3.3%) were positive for HCV-RNA in PBMCs, identifying a subset of subjects with potential occult HCV infection. We could determine the HCV type for 8 of the 9 patients finding type 1a (3 patients), type 1b (2 patients), and type 2a (3 patients).

**Conclusions:**

The results of this study show evidence that occult HCV infection may occur in a population unselected for hepatic disease. A potential risk of HCV infection spread by subjects harbouring occult HCV infection should be considered. Design of prospective studies focusing on the frequency of infection in the general population and on the clinical evolution of occult HCV infection will be needed to verify this unexpected finding.

## Introduction

Occult Hepatitis C virus (HCV) infection has been defined [1]–[5] as a pathological entity displaying different clinical features from typical HCV infection. HCV infection is routinely diagnosed and monitored by the detection of HCV antibodies and/or HCV-RNA in plasma or serum. Subjects affected by occult HCV infection test negative for HCV-RNA in serum, but they are HCV-RNA positive in liver biopsies and may display abnormal values of liver enzymes. Occult HCV infection may occur under two different clinical situations: patients may show either negativity for both serum HCV antibodies and HCV-RNA with abnormal liver function tests, or positivity for HCV antibodies and no detectable serum HCV-RNA with normal liver enzymes, due to clearance of infection [5]–[8]. Furthermore, the presence of low levels of HCV genomes (silent HCV infections) in different pathological setting was reported, mainly in subjects with a previous history of HCV related disease [6], [9]–[12]. Among patients with cryptogenic chronic hepatitis, those with occult HCV infection had more liver inflammation and fibrosis than those without occult HCV infection [1]. In occult HCV patients, the presence of HCV-RNA was also identified in peripheral blood mononuclear cells (PBMCs) [1], [2], [13], which represent alternative extrahepatic site of HCV replication [2], [4], [14], [15] proposed as a source of recurrent HCV infection after liver transplantation [2], [16], [17]. Different immune cell subsets (e.g. CD4+ and CD8+ T lymphocytes, B cells and monocytes) can be HCV infected. HCV may be also confined to a specific immune cell subtype with risk of low analytical HCV-RNA detection. New technologies employing multiple mitogens stimulation has been improved to avoid false negative results [13], [18], [19]. Although the mechanism of HCV replication is not completely understood, viral replication is assumed to involve the synthesis of a negative-strand RNA molecule (antigenomic HCV-RNA) that acts as a template for production of a positive-strand RNA molecule (genomic HCV-RNA) [2], [15], [20]. The detection of the antigenomic HCV-RNA can, therefore, be assumed to be an indication of active viral replication. Both the genomic and the antigenomic HCV-RNA strands have been detected in PBMCs of patients with occult HCV infection [2], [21], supporting the hypothesis that HCV is able to replicate in these cells.

In the frame of a case-control study nested in the EPIC (European Prospective Investigation into Cancer and Nutrition) Italy cohort [22], [23], designed to evaluate the etiological role of viruses (HCV included) in the occurrence of Non-Hodgkin's Lymphoma, we were surprised to find that 7/176 of the control subjects displayed features characteristic of occult HCV infection (Richiardi et al submitted). These subjects were disease free when enrolled and tested negative for both HCV antibodies and plasma HCV-RNA, but they resulted positive for HCV-RNA in the PBMCs. Therefore, occult HCV infection may occur in the general population apparently disease free. The presence of viral replicative ability in PBMCs could represent a potential risk of HCV spread through transfusion, haemodialysis [4], [24], or of liver disease evolution (e.g. liver neoplasia) in occult HCV infected subjects [4], [25].

To support the results obtained in control samples from the EPIC Italy cohort study (Richiardi et al. submitted), we analysed two additional independent series for detection of HCV antibodies and HCV-RNA both in plasma and in PBMCs.

## Results

All subjects in the study had normal levels of ALT (range from 5 to 17 IU/L) and AST (range from 5 to 25 IU/L) and resulted negative for HCV antibodies and viremia. All RNA extracted samples resulted positive at the b-actin amplification, confirming adequacy for the HCV PCR analyses. Considering all study series (“EPIC Italy”, “Turin Case-Control Bladder Cancer”, “Cervical Cancer Screening”), we found that 9 out of the 276 (3.3%; 95% CI: 1.5–6%) subjects testing negative for HCV antibodies and viremia were positive for HCV-RNA in their PBMCs. HCV-RNA positivity was confirmed by direct sequencing matching the 5′-UTR region of HCV genome according to the Blast sequence. As summarized in Table 1, 7/176 (4.0%) subjects in the EPIC Italy series and 2/60 (3.3%) subjects in the Cervical Cancer Screening series, were positive for HCV-RNA in PBMCs. All (40/40, 100%) subjects in the Turin Case-Control Bladder Cancer series tested negative. HCV genotypes were determined for 8 of 9 specimens resulting HCV type 1a, 1b and 2a (Table 2), one sample was not genotyped since a very weak PCR fragment was detected.

**Table 1:** HCV detection in three series of subjects.

Subjects	Number of subjects	HCV antibodies (%)	HCV –RNA plasma (%)	HCV –RNA PBMCs (%)^a^
**All series**				
All subjects	276	0 (0)	0 (0)	9 (3.3%)
Men (age range: 36–65 years)	111	0 (0)	0 (0)	3 (2.7%)
Women (age range: 39–76 years)	165	0 (0)	0 (0)	6 (3.6%)
**EPIC Italy-cohort series**				
All subjects	176	0 (0)	0 (0)	7 (4.0%)
Men (age range: 36–65 years)	71	0 (0)	0 (0)	3 (4.2%)
Women (age range: 39–76 years)	105	0 (0)	0 (0)	4 (3.8%)
**Turin Case-Control Bladder Cancer series(controls)**				
Men (age range: 42–66 years)	40	0 (0)	0 (0)	0 (0%)
**Cervical Cancer Screening series**				
Women (age range: 46–61 years)	60	0 (0)	0 (0)	2 (3.3%)

a PBMCs, peripheral blood mononuclear cells.

**Table 2:** Characteristic of subjects showing occult HCV.

Patient	Sex	Age	ALT/AST*	HCV antibody	HCV-RNA plasma	HCV-RNA PBMCs	HCV Genotyping
1	M	51	−/−	−	−	+	1a
2	M	60	−/−	−	−	+	n.d.
3	F	46	−/−	−	−	+	1b
4	F	52	−/−	−	−	+	1b
5	F	56	−/−	−	−	+	2a
6	M	41	−/−	−	−	+	2a
7	F	54	−/−	−	−	+	2a
8	F	55	−/−	−	−	+	1a
9	F	47	−/−	−	−	+	1a

* : −/− indicates normal values below 50 IU/L and 35 IU/L for men and women respectively for ALT, and below 40 IU/L and 32 IU/L for men and women respectively for AST determination.

n.d.: the amplification resulting in a very weak fragment and sequence analysis did not succeed.

## Discussion

At present, occult HCV infection has been described in relation to hepatic diseases, and data on the frequency of HCV-RNA in the PBMCs of the general population are unknown.

We identified subjects apparently free of liver disease, since testing normally for liver enzymes and negative for serological HCV markers, which unexpectedly showed potential occult HCV infection features.

Negative serological results were unlikely due to low sensitivity, as the tests employed to detect HCV antibodies and circulating virus have been extensively reported to have high reliability, with a 98.8% sensitivity and a 100% specificity [1]. If these values of specificity and sensitivity are applied to a prevalence of 2.7% of detection of HCV antibodies found in the Italian population [26] the Negative Predictive Value (NPV according to Bayes theorem) is 0.99966, and the probability that 9 or more of the 276 negative results were false negatives is virtually zero (4.1^−15^, Exact Binomial Probability).

Despite the most prevalent HCV genotype in Italy [26] is 1b, interestingly we identified the presence of different HCV genotypes (1a, 1b, 2a) in the PBMCs of our study population. Similar results were as well observed in other series of subjects considered to be clinically and serologically cleared of HCV infection [6].

The unexpected result of this study raises the issue of occult HCV infection frequency in the general population and its potential implications on the safety of medical procedures and the onset of liver disease. Subjects with occult HCV infection may not harbor detectable circulating virions, but they could be still potentially infectious, as PBMCs have been demonstrated to be permissive for viral replication [4].

The potential risk for HCV spread from occult HCV subjects through blood transfusions and hemodialysis units should be considered [27]. Despite advances in the screening procedures for infectious agents in blood from donors, the risk of transmission via transfusion of viral, bacterial, and protozoal infections, as well as from newly emerging diseases, remains [28]. Despite approaches to leukocyte-related risk reduction as the leukodepletion [28]–[31], the efficacy in reducing the risk of transmitting infectious agents is still under discussion for some viruses (cytomegalovirus, human herpesvirus-8, Epstein–Barr virus, human T-cell leukemia/lymphoma virus) and prions [28], [29]. Therefore, those donors who carry HCV in their PBMCs may be overlooked by current screening strategies [4].

Moreover, since it has been observed that very low-level of HCV-RNA may persist in the PBMCs for many years representing a potential risk for the recurrence of infection, the clinical potential in hepatocarcinogenesis should be evaluated [9], [32]. With this study we could not deeply evaluate the implications of persistent HCV-RNA in PBMCs on liver disease evolution since no information was available about the follow-up of the studied subjects. Neither analysis about the viral load were performed due to limited amount of sample. As well, limited amount of both plasma and PBMCs, and PBMCs storage in no vital conditions, did not allow performing of techniques developed in recent years as PBMCs mitogen stimulation [6] and ultracentrifugation which is mainly performed with at least 2 ml of plasma [33]. However, the fact that we found HCV RNA positivity in PBMCs despite the limited amount may result in an underestimate of the phenomenon, which further strengthens the relevance of our results. Whenever we had found a HCV RNA positivity after ultracentrifugation of plasma from the same subjects, the final result of occult HCV would not be altered [7].

The results of this study show evidence that occult HCV infection may occur in a population unselected for hepatic disease. A potential risk of HCV infection spread by subjects who unknowingly may carry occult HCV infection and of liver disease onset should be considered.

Design of prospective studies focusing on the prevalence and on the clinical evolution of occult HCV infection in the general population will be needed to verify this unexpected finding, as well as the analysis of HCV-RNA replication potential, the identification of HCV genotypes involved in occult HCV infection, and the evolution of liver disease.

## Materials and Methods

### Ethic Statement

Ethic statement was not required for this specific study. All participants recruited, controls included, donated blood samples in previous epidemiological studies, following written informed consent given to principal investigators. These blood samples were re-used, since previous ethical approval included enabling other non-specified tests to be carried out on the biological samples supplied.

### Study Population

The present study was performed including a total of 276 subjects (111 men and 165 women) as sample of the general population. All subjects aged between 40 and 65, were apparently liver disease free at recruitment, and were enrolled in the frame of three different epidemiologic studies previously approved by local ethic committees: EPIC Italy cohort (protocol number: 96/01) [22], [23], Turin Case-Control Bladder Cancer Study (protocol number: CEI/174)[34], Cervical Cancer Screening (registration number: ISRCTN81678807) [35] (Table 1).

All participants in the studies, following written informed consent given to principal investigators, donated a blood sample, which was collected and fractioned (red blood cells, buffy-coat, serum and plasma) on the day of enrolment and snap frozen in liquid nitrogen or at −80°C.

EPIC Italy cohort series: The European Prospective Investigation into Cancer and Nutrition (EPIC) included healthy subjects enrolled in 10 European countries. The EPIC Italy cohort involved 5 centres (Turin, Varese, Florence, Naples, and Ragusa) with a total of 47,000 subjects enrolled between 1992 and 1998. In the present study, we included 176 subjects randomly selected as controls for the Non-Hodgkin's Lymphoma study (Richiardi et al. submitted) from the eligible members of the EPIC Italy cohort: 39 from Turin, 74 from Varese, 42 from Florence, 11 from Naples, and 10 from Ragusa. These controls were matched to the corresponding lymphoma case on sex, centre, age (within 5 years), and period of collection of the blood sample (within 90 days). They were a sample of the general population, although blood donors and attendants to screening programs were over-sampled. Detailed information for each volunteer about diet and life-style habits, anthropometric measurements and residential and occupational history was collected.

Turin Case-Control Bladder Cancer Study series: Hospital-based case-control investigations at two urology departments in S. Giovanni Battista Hospital in Turin, Italy. Subjects with newly diagnosed bladder cancer in the Turin metropolitan area were recruited as cases. Subjects with benign diseases, mainly prostatic hyperplasia and cystitis (all newly diagnosed), and patients treated at the medical and surgical departments for hernias, vasculopathies, diabetes, heart failure, asthma or other benign diseases (none was represented in >10%), were recruited from the medical and surgical departments of the same hospital as controls. Patients with cancer other than bladder, liver or renal disease, and smoking-related conditions were excluded. In the present study were included 40 men (aged between 40 and 65) consecutively recruited as controls between 2001 and 2003.

Cervical Cancer Screening series: Italian project designed in the frame of Cervical Cancer Screening Programmes to study papillomavirus infection, frequency, prevalence, and implication in cervical lesions progression. In the present study, we included 60 women (aged between 40 and 65) consecutively recruited in Turin between 2002 and 2004 which gave consent to donate a blood sample.

### Liver Enzymes Detection

Quantitative levels of alanine aminotransferase (ALT) and aspartate aminotransferase (AST) in plasma were evaluated by using an UV test on Roche automated clinical chemistry analyzer (Cobas, Roche/Hitachi Modular analyzers, North America, USA). This method has been standardized according to the original International Federation of Clinical Chemistry (IFCC) formulation. The expected normal values according to IFCC were up to 50 IU/L and up to 35 IU/L for men and women respectively for ALT, and up to 40 IU/L and up to 32 IU/L for men and women respectively for AST determination. The analytical sensitivity is 4 IU/L for both the determinations, and the measuring range is 4–600 IU/L and 4–800 IU/L for ALT and AST respectively.

### HCV Antibodies Research

Detection of HCV antibodies was performed by a commercial enzyme immunoassay test of third-generation (ORTHO HCV 3.0 – commercialized by Dia Sorin, Saluggia, Italy) following the manufacturer instructions. Samples showing optical densities above the cut off value were considered positive. The sensitivity and specificity of the test were estimated of 98.8% and 100% respectively [1], [36].

### HCV RNA Extraction and Detection

Total RNA was isolated from 150 µL of buffy-coat and 150 µL plasma using SV total RNA Isolation system (Promega, Madison, Wi, USA) and EXTRAgene (Amplimedical, Turin, Italy) respectively, following the manufacturer instructions. In 150 µL of buffy-coat there was a range between 10^6^ and 10^7^ cells: they were washed three times by using SV RNA Red Blood Cell Lysis Solution (Promega, Madison, Wi, USA) in order to avoid contaminants carry over. Adequacy of RNA extraction was evaluated by retro-transcription of 5 µL by the Reverse Transcription System (Promega) to cDNA and amplification of a 171 bp fragment of the b-actin gene (primer forward 5′-CCCCGCGAGCACAGA-3′ and reverse 5′-CCACGATGGAGGGGAAGAC-3′) according to Lossos et al, 2003 [37]. Detection of HCV-RNA was performed by a commercial RT-nested PCR system (Alfa Wasserman, Milan, Italy) using primers targeting the 5′UTR region of the HCV genome, reagents and controls included in the kit. According to the manufacturer instructions, five microliters of extracted RNA were in single step reverse transcribed and amplified as follows: 30 min at 42°C (retrotranscription); 5 min at 95°C; 25 s at 94°C, 20 s at 50°C, 40 s at 72°C for 30 cycles; and a final extension of 5 min at 72°C. Three microliters of RT-PCR reaction were added to the second step (nested) PCR and amplified for other 30 cycles, using the same cycling conditions of the first step. Amplicons were analysed on 2% agarose gel stained with ethidium bromide and visualized by ultraviolet transillumination. The presence of a 187 bp amplification fragment indicate HCV-RNA positivity in the sample. Synthetic HCV-RNA corresponding to 5′UTR region, HCV negative human serum and RNA-free sample were included as positive and negative controls respectively in each PCR run. The sensitivity of this test was up to 50 genome equivalents (6 IU/ml). All positive samples were repeated for confirmation in different runs of PCR amplification to exclude contamination occurrence.

We used plasma rather than serum because prior RT-PCR studies showed a higher sensitivity when the analyses were performed with plasma [15], [38].

### HCV Genotyping

The positive PCR products of the 5′UTR region were excised from the gel and purified with the “PCR Clean-up Gel Extraction Kit” (Macherey-Nagel, Dueren, Germany). Direct sequencing was carried out using the Big Dye Terminator Cycle Sequencing Ready Reaction Kit (Applera, Monza, Italy), and sequence analysis was performed with the ABI 310 automated capillary system (Applera, Monza, Italy), following the manufacturer instructions. Sequences were analyzed with the BLAST Software (http://www.ncbi.nlm.nih.gov/BLAST). All HCV genotypes were defined with a query coverage ≥98%, analyzing at least 170 nucleotides of the target sequence.
